# Can MRI Biomarkers Predict Triple-Negative Breast Cancer?

**DOI:** 10.3390/diagnostics10121090

**Published:** 2020-12-15

**Authors:** Giuliana Moffa, Francesca Galati, Emmanuel Collalunga, Veronica Rizzo, Endi Kripa, Giulia D’Amati, Federica Pediconi

**Affiliations:** Department of Radiological, Oncological and Pathological Sciences, Sapienza—University of Rome, 00161 Rome, Italy; francesca.galati@uniroma1.it (F.G.); e.collalunga@libero.it (E.C.); veronica.rizzo0388@gmail.com (V.R.); endi.kripa@uniroma1.it (E.K.); giulia.damati@uniroma1.it (G.D.); federica.pediconi@uniroma1.it (F.P.)

**Keywords:** triple-negative breast cancer, 3 T MRI, DWI, breast cancer prognostic factors

## Abstract

The purpose of this study was to investigate MRI features of triple-negative breast cancer (TNBC) compared with non-TNBC, to predict histopathological results. In the study, 26 patients with TNBC and 24 with non-TNBC who underwent multiparametric MRI of the breast on a 3 T magnet over a 10-months period were retrospectively recruited. MR imaging sets were evaluated by two experienced breast radiologists in consensus and classified according to the 2013 American College of Radiology (ACR) BI-RADS lexicon. The comparison between the two groups was performed using the Chi-square test and followed by logistic regression analyses. We found that 92% of tumors presented as mass enhancements (*p* = 0.192). 41.7% of TNBC and 86.4% of non-TNBC had irregular shape (*p* = 0.005); 58.3% of TNBC showed circumscribed margins, compared to 9.1% of non-TNBC masses (*p* = 0.001); 75% of TNBC and 9.1% of non-TNBC showed rim enhancement (*p* < 0.001). Intralesional necrosis was significantly associated with TNBC (*p* = 0.016). Rim enhancement and intralesional necrosis risulted to be positive predictors at univariate analysis (OR = 29.86, and 8.10, respectively) and the multivariate analysis confirmed that rim enhancement is independently associated with TNBC (OR = 33.08). The mean ADC values were significantly higher for TNBC (*p* = 0.011). In conclusion, TNBC is associated with specific MRI features that can be possible predictors of pathological results, with a consequent prognostic value.

## 1. Introduction

Breast cancer is a heterogeneous disease with different morphologic features, clinical course and response to treatment. As a consequence, personalized management is required [1,2]. In addition to traditional parameters, such as tumor size, histological grade and lymph node status, molecular markers have been introduced into the patient’s care routine [3]. At present, surrogate definitions of breast cancer subtypes are based on immunohistochemical measurements of the expression of estrogen receptor (ER), progesterone receptor (PgR), human epidermal growth factor receptor 2 (HER2), with in situ hybridization confirmation where appropriate, and on the assessment of the proliferation index. According to immunohistochemistry, five clinico-pathologic breast cancer subtypes were identified: luminal A-like, luminal B-like HER2 negative, luminal B-like HER2 positive, HER2 positive, and triple-negative [4].

Triple-negative breast cancer (TNBC), characterized by ER, PgR and HER2 negativity, is commonly used as a surrogate for intrinsic basal-like subtype and accounts for 10–20% of invasive breast cancers [5]. TNBC is a biologically and clinically aggressive tumor (with an increased rate of local recurrence and distant metastases, compared to other breast cancer subtypes [5]), characterized by early onset (usually <50-year old women) and frequent association with BRCA 1 mutation [5,6]. In spite of the aggressiveness, TNBC can mimic benign lesions at conventional breast imaging, lacking the typical malignant features of cancer. On mammography, TNBC usually presents as a mass, with circumscribed margins (8–43% of cases) and without associated calcifications (89–100% of cases) [7,8]. On ultrasound examination (US), TNBC often appears as a regular-shaped mass with well-circumscribed margins (21–27% [8]), parallel orientation and posterior acoustic enhancement (24–41% [8]) [9]. For what concerns multiparametric magnetic resonance imaging (MP-MRI), previously published papers have described the most typical features of TNBC that comprised mass-shaped lesions, rim enhancement, and intra-tumoral hyperintensity at T2-weighted sequences [7,10,11,12,13]. Moreover, preliminary researches have demonstrated that TNBC is more likely to have a higher apparent diffusion coefficient (ADC) value on diffusion-weighted imaging (DWI) than other breast cancer subtypes [14,15].

In the precision medicine era, it seems particularly important for every breast radiologist to suspect and ultimately non-invasively predict histological results of TNBC, considering the particularly aggressive behavior of this diagnostic misleading subtype. As far as the authors know, there are few studies in literature that investigated the differences between MRI features of TNBC and non-TNBC and were conducted mostly on 1.5 T scanners, not including functional techniques, such as DWI [10,14,16,17,18,19,20,21]. Therefore, the aim of this study was to investigate MRI features of TNBC compared with non-TNBC on a 3 T scanner, to increase existing knowledge about TNBC, including elements of radiological-pahological correlation.

## 2. Materials and Methods

### 2.1. Study Population

The study was conducted according to Good Clinical Practice guidelines and obtained the approval of our institutional review board (#0525032019, approved 25 March 2019). The requirement for informed consent was waived because of the retrospective nature of the study.

From April 2017 to January 2018, 162 patients with newly diagnosed biopsy-proven breast cancer who underwent MP-MRI of the breast at the time of diagnosis were included in this study.

Core needle biopsies (CNB) were performed under US guidance by two experienced breast interventional radiologist using a 12 MHz linear probe (Affiniti 70 G; Philips, Amsterdam, Netherlands) and a 14-gauge semi-automatic biopsy needle (Precisa; HS Hospital Service SpA, Aprilia, Italy).

Exclusion criteria included; the inability to complete MRI examination (n = 5); previous history of breast cancer or recurrent disease (n = 21); ongoing neoadjuvant chemotherapy (NACT) or other cancer treatments (n = 16); presence of breast implants (n = 18); CNB performed less than 14 days before MRI, to eliminate possible bias due to the diagnostic procedure such as the presence of a voluminous post-biopsy hematoma (n = 15); complete histological data not available (n = 10).

Out of the remaining 77 patients, 26 with histological diagnosis of TNBC were used for the evaluation. The group included women aged between 35 and 60 years, affected by invasive breast carcinoma. A control group, composed of a similar number of patients of the same age with histologically proven non-TNBC and who underwent breast MP-MRI during the same period, was randomly included (n = 24).

Clinical data were obtained from medical records. Patient data was collected using Excel 2011 (Microsoft Corporation, Redmont, WA, USA).

### 2.2. MRI Technique

All breast MRI examinations were performed on a 3 T magnet (Discovery MR 750; GE Healthcare, Chicago, IL, USA) with a dedicated 8-channel breast coil compatible with parallel imaging, and patients in a prone position. Breast MRI protocol included: axial pre-contrast 2D FSE T2-weighted fat-suppressed sequence (repetition time [RT] = 9000–11,000 ms, echo time [ET] 119–120 ms, matrix = 512 × 224, slice thickness = 3–5 mm, field of view [FOV] = 35 × 35 cm, NEX = 1, scan time = 130 s), axial pre-contrast diffusion-weighted echo-planar imaging (DWI-EPI) sequence (RT = 4983–5314 ms, ET = 58 ms, matrix = 150 × 150, slice thickness = 3–5 mm, FOV = 350 × 350 mm, NEX = 2–2–4, scan time = 230 s), axial dynamic three-dimensional (3D) spoiled gradient-echo T1-weighted fat-suppressed sequences (flip angle = 15°, RT = 8 ms, ET = 4 ms, matrix = 512 × 256, slice thickness = 1.40 mm, FOV = 380 × 380 mm, NEX = 1) and sagittal 3D spoiled gradient-echo post-contrast T1-weighted sequence.

Fat suppression of T2-weighted sequences was based on a three-point Dixon technique (IDEAL). DWI-EPI sequences comprised b-values of 0, 500 and 1000 s/mm^2^ and the corresponding apparent diffusion coefficient (ADC) maps were calculated automatically. Axial dynamic 3D T1-weighted fat-suppressed sequences (VIBRANT) were performed before and four times after contrast agent administration (total acquisition time of 120 s). Post-contrast T1-weighted images were acquired after the administration of 0.1 mmol/kg (0.2 mL/kg) gadolinium-based contrast agent (Gadoteridol—Prohance 279.3 mg/mL; Bracco Imaging Italia S.r.l., Milano, Italy) at a rate of 2 or 3 mL/s. Gadoteridol was power-injected through a peripheral venous access (22 gauge) and was followed by a 20-mL saline flush. Post-processing subtraction images were obtained for all examinations.

Imaging of premenopausal women was performed between the 7th and the 14th day of the menstrual cycle, according to current guidelines [22].

### 2.3. MR Imaging Evaluation

MRI images evaluation was performed retrospectively by two experienced breast radiologists, with 15 and 7 years of experience, respectively. The readers, blinded to clinical and histopathological information, evaluated all the MR imaging sets in consensus at the system console, using the automated software available. The evaluation was performed using all images available and classified according to the 2013 American College of Radiology (ACR) Breast Imaging Reporting and Data System (BI-RADS) lexicon [23]. All the lesions were measured and size in mm was reported. The presence of intralesional necrosis and perilesional edema was assessed visually on pre-contrast fat-suppressed T2-weighted images as areas of high signal intensity (as high as that of water) localized within the lesion, and around or posteriorly to the lesion, respectively. Moreover, T2-weighted signal intensity of each lesion was evaluated visually and classified as hypointense (when lower than surrounding glandular tissue), isointense (same intensity as glandular tissue), and hyperintense (when higher than glandular tissue) on the basis of the predominant signal intensity of the lesion. Hyperintense regions were qualitatively evaluated on high b-value images (b = 1000 s/mm^2^). ADC values of lesions were obtained drawing manually a two-dimensional (2D) region of interest in the center of the area of restricted diffusion on ADC maps.

The presence of possible axillary lymphadenopathies (characterized by: size > 1 cm, round shape, loss of the fatty hilum, cortical thickening) was assessed on post-contrast fat-suppressed T1-weighted sequences.

### 2.4. Histopathological Analysis

All the specimens were evaluated according to standardized protocols by a pathologist with more than 20 years of experience. The specimens were fixed in 10% formalin for 6 to 8 h, then processed to obtain paraffin blocks subsequently cut in 5-μm-thick slices and stained with hematoxylin-eosin. Tumors were classified following the World Health Organization Classification and graded according to the Nottingham Histologic Score. The immunohistochemical analysis was carried out using mouse monoclonal antibodies anti-estrogen receptor (ER) alpha (6F11; Novocastra Laboratories Ltd., Newcastle upon Tyne, UK) and anti-progesterone receptor (PgR-312; Novocastra Laboratories Ltd., Newcastle upon Tyne, UK). HER2 evaluation was performed using a semiquantitative immunohistochemical assay (HercepTest; Dako Agilent, Santa Clara, CA, USA); the intensity of HER2 membrane staining was scored as 0, 1+, 2+ or 3+. In case of equivocal result (2+) fluorescence in situ hybridization for HER2 gene amplification was performed, according to the 2013 American Society of Clinical Oncology/College of American Pathologists guidelines [24]. Proliferation index was determined using anti-Ki-67 monoclonal antibody MM1 (Novocastra Laboratories Ltd., Newcastle upon Tyne, UK). The Ki-67 value was expressed as the percentage of tumor cells showing nuclear staining. Considering the immunohistochemical features, tumors were classified as luminal A-like, luminal B-like HER2-negative, luminal B-like HER2-positive, HER2-positive, and triple-negative, according to the 2013 St. Gallen Consensus Conference [4].

### 2.5. Statistical Analysis

The Kolmogorov-Smirnov Z test was performed to assess the normality of the distribution for all the variables tested. Continuous normal variables were expressed as mean ± standard deviation (SD) while continuous non-normal variables were expressed as median and range. Categorical variables were expressed as percentages. A comparison of categorical variables was performed using the Chi-square test with Yates correction, while the Bonferroni correction was used for post-hoc Chi-square analysis. Univariate and multivariate logistic regression analyses were performed to identify the predicting value of imaging-derived features associated with TNBC. The statistical analyses and the graphs plotting were performed using the MedCalc Software version 8.0 (MedCalc Software, Mariakerke, Belgium).

*p*-values < 0.05 were considered statistically significant. All *p*-values were calculated using a two-tailed significance level.

## 3. Results

A total of 50 patients were included in this study, 26 affected by TNBC (group 1) and 24 affected by non-TNBC (group 2). Patients’ age ranged from 35 to 60 years (mean age = 50.5, SD = 7.2). The mean age at diagnosis was 49.9 (SD = 8.1) in group 1, and 50.9 (SD = 6.6) in group 2, respectively. No statistical association was found between age and TNCB subtype in our study population (*p* = 0.839).

Median lesion size at MR imaging was 21.5 mm (range 6–60 mm). 57.7% of TNBCs were ≥2 cm, while 42.3% were smaller. Among non-TNBCs, only 45.8% measured ≥2 cm. No statistical association was found between tumor size and TNBC subtype (*p* = 0.308), even if TNBC were larger than non-TNBC on average.

In the MRI examination most tumors in both groups appeared as mass enhancement: 92.3% in group 1 (TNBC), and 91.7% in group 2 (non-TNBC), respectively (*p* = 0.192). Most masses were characterized by irregular shape (41.7% of TNBCs, and 86.4% of non-TNBCs, respectively), with a statistically significant difference in frequency between the two groups (*p* = 0.005). For what concerns margins, TNBC masses showed circumscribed margins in 58.3% of patients, while non-TNBC masses only in 9.1% of cases (*p* = 0.001). Internal enhancement characteristics were classified as rim enhancement in 18 patients (75%) of group 1 (TNBC) and only 2 patients (9.1%) of group 2 (non-TNBC). Rim enhancement confirmed to be one MRI feature that is significantly associated with TNBC subtype (*p* < 0.00001) in our study population.

Only 4 patients (8% of the entire study population) showed non-mass enhancements at breast MRI: 2 (7.7%) with TNBC (1 segmental and 1 regional distribution) and 2 (8.3%) with non-TNBC (2 regional), respectively.

TNBCs showed T2-signal hyperintensity in 6 cases (23.1%) and hypo-isointensity in 20 cases (76.9%), while non-TNBCs were T2-hyperintense in 2 cases (8.3%) and hypo-isointense in 22 cases (91.7%) (*p* = 0.301). Additional tumor features detectable on T2-weighted sequences, such as intralesional necrosis, resulted significantly associated with the TNBC subtype (*p* = 0.016) in our study population, while no association was found between TNBC and peritumoral edema (*p* = 0.880).

13 (50%) TNBCs and 5 (20.8%) non-TNBCs were multifocal or multicentric (*p* = 0.064) and 16 (61.5%) of group 1 (with TNBC) and 10 (41.7%) of group 2 (with non-TNBC) had at least one axillary or internal mammary lymphadenopathy. No statistically significant association was found between the detection of loco-regional lymphadenopathies at MRI and TNBC subtype (*p* = 0.262) in 26 patients (52% of total population).

Further details about MRI features associated with TNBC and non-TNBC are shown in Table 1.

The univariate analysis proved that not circumscribed margins are a negative predictor of TNBC (OR = 0.03, CI 95% = 0.01–0.17, *p* = 0.0001), while rim enhancement and the presence of intralesional necrosis are positive predictors (OR = 29.86, CI 95% = 5.53–161.35, *p* = 0.0001 and OR = 8.10, CI 95% = 1.56–41.72, *p* = 0.01, respectively). The multivariate analysis confirmed that the presence of rim enhancement is independently associated with TNBC (OR = 33.08, CI 95% = 1.59–687.55, *p* = 0.02) while not circumscribed margins are not (OR 0.03, CI 95% = 0.002–0.34, *p* = 0.005) (see Table 2).

The mean ADC values were 1.04 × 10^−3^ mm^2^/s (SD = 0.2) for TNBCs and 0.92 × 10^−3^ mm^2^/s (SD = 0.1) for non-TNBCs (*p* = 0.011).

## 4. Discussion

**Figure 1:**
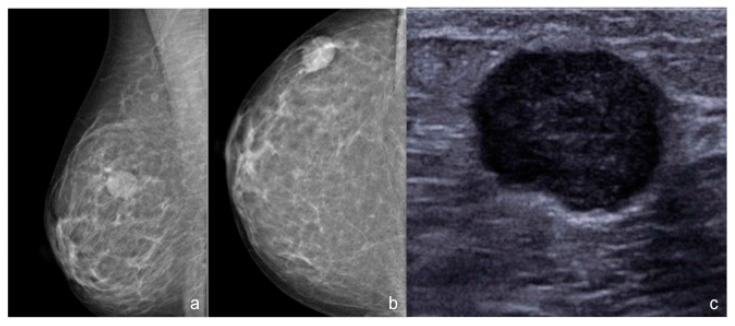
55-year-old woman with a palpable nodule in the upper outer quadrant of the right breast. (**a**,**b**) Standard mammography of the right breast (ACR B) shows a corresponding round high-density mass, with circumscribed margins. (**c**) Targeted US scan detects a corresponding round hypoechoic mass with slightly microlobulated margins. The histopathological analysis after CNB revealed a TNBC.

**Figure 2:**
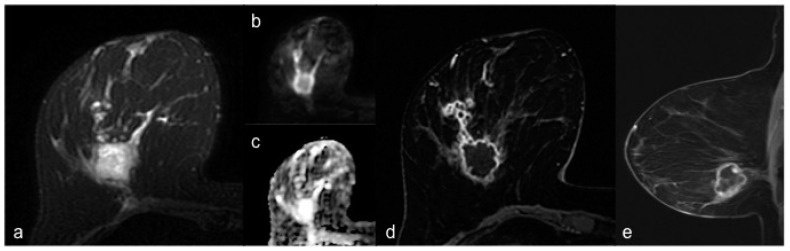
Breast MRI of a 53-year-old woman with TNBC of the right breast. (**a**) Axial fat-suppressed T2-weighted image shows a hyperintense round mass with irregular margins in the lower outer quadrant of the right breast. (**b**) Axial DWI image (b value = 1000 s/mm^2^) shows peripheral high signal intensity and central hypointensity. The mass appears homogeneously hyperintense in the corresponding ADC map (**c**). (**d**) Axial and sagittal (**e**) post-contrast T1-weighted images show a corresponding irregular round mass with rim enhancement and some small foci of enhancement connected posteriorly by thin striae.

Triple-negative tumor is an aggressive subtype of breast cancer with peculiar features in terms of biology and response to therapy. As known, TNBC is highly chemosensitive and cannot benefit from endocrine or targeted therapy, unlike other subtypes of breast cancer. For this reason, NACT represents nowadays the treatment of choice in this group of tumors [2,5,25]. It is also well-known that TNBC has frequently benign-like features at conventional breast imaging (Figure 1) and that it presents specific associated features at MRI (Figure 2) more often than other subtypes of breast cancer. Moreover, a preliminary study has demonstrated that TNBC is prevalent among patients with marked background parenchymal enhancement [26]. Therefore, the aim of this study was to investigate MRI features of TNBC compared with non-TNBC, to possibly predict histopathological results on the basis of MRI biomarkers, considering that, as far as the authors know, there are few studies based on the comparison between MRI features of TNBC and non-TNBC and that the great majority of them were conducted at 1.5 T scanners and did not include functional techniques such as DWI [10,14,16,17,18,19,20,21].

**Figure 3:**
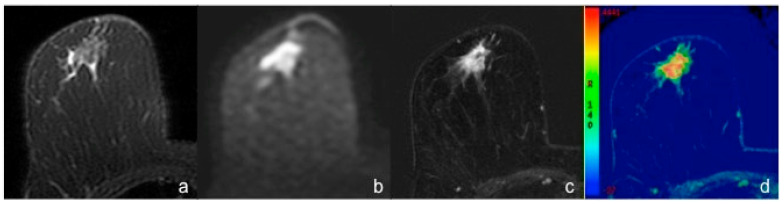
Breast MRI of a 57-year-old woman with IDC of the right breast (Luminal B). (**a**) Axial fat-suppressed T2-weighted image shows a hypointense irregular mass with spiculated margins and some hyperintense peripheric areas consistent with perilesional edema, in the retroareolar region. (**b**) Axial DWI image (b value = 1000 s/mm^2^) shows the high signal intensity of the mass, corresponding to a restricted diffusion area. (**c**) Axial early post-contrast T1-weighted image and the corresponding perfusion color map (**d**) show a 16 mm irregular mass with spiculated margins and heterogeneous enhancement.

In our study, the majority of TNBCs appeared as mass enhancements characterized by circumscribed margins at MRI examination. Moreover, 58.3% of TNBCs in our sample were regular shaped masses (round or oval). These results confirm existing data in literature [10,11,12,14]. On the contrary, irregular shape was significantly more frequently associated with non-TNBC (Figure 3).

These features can be explained by the typical non-infiltrative growth pattern of TNBC, characterized by “pushing borders” [27], with good demarcated and “rolled-back” margins [28,29]. Considering the imaging and pathological features of these tumors, Yang et al. [30] suggested the existence of a more rapid pattern of carcinogenesis for TNBC, compared to other subtypes of breast cancers, that leads directly to invasive cancer, without major in situ components or precancerous stages. This theory could explain the lack of calcifications on standard mammography and the low incidence of ductal carcinoma in situ (DCIS) in TNBC. In this study 100% of TNBCs were pure invasive ductal carcinomas (IDC) without cases of associated DCIS; this result is higher than data in literature (80–90% of TNBCs are IDC [5,31]), but it could be explained by the relative small number of the sample. Moreover the rapid growth of TNBC, that usually presents as an interval cancer [5], could be responsible for the absence of the desmoplastic reaction that is typically associated to breast cancer and is responsible for the infiltrating appearance [21,32].

Our study confirmed that rim enhancement is a positive predictor of TNBC subtype at histological examination, and that it is independently associated with TNBC. These results are consistent with what previously reported in literature [10,12,17,33]. In this study internal enhancement characteristics were classified as rim enhancement in 75% of TNBCs, compared to 80% reported by Uematsu et al. [33], 76% by Dogan et al. [12], 68% by Angelini et al. [17], and 57% by Sung et al. [10]. Rim enhancement on DCE-MRI is an established finding of malignant breast lesions, and it is known to be associated with increased angiogenesis and vascular endothelial growth factor expression and negative expression of estrogen and progesterone receptors [34,35]. Moreover, several research studies have shown that recurrence occurred more frequently in rim-enhancing TNBC [34,36,37].

The presence of intralesional necrosis was the other positive predictor of TNBC subtype in this study, in accordance with literature [16,33]. In this study only 23% of TNBCs showed high T2-signal intensity, compared to 25–84% reported in previous studies [7,10,14,17,20,33,34]; however 75% of the hyperintense tumors in both groups (6/8) were TNBC. The reason behind this variability remains unclear, but it could be mainly explained by the fact that in the present study the evaluation of T2-signal intensity was based on the predominant signal intensity of each lesion; as a consequence, lesions with only small areas of T2-hyperintensity, possibly related to intralesional necrosis, were not classified as T2-hyperintense. Anyway it should be remembered that T2-hyperintensity is due to a combination of abundant cytoplasm, edematous stroma and necrosis [17,33] and that histologic correlation was not investigated, except in one mentioned study [33].

Another breast tumor-associated feature detectable on T2-weighted sequences is peritumoral edema, which was demonstrated to correlate with biologically aggressive non-luminal breast cancers [38,39]. However, no association was found between TNBC and peritumoral edema in this study. This result can be explained by the larger tumor size of TNBCs included in those studies compared to ours and by the small number of patients.

TNBC tumor size is usually larger than non-TNBC on MRI at diagnosis [27]. In this study, TNBC were larger than non-TNBC on average, even if no statistical association was found between tumor size and TNBC subtype. This result is presumably explained once again by the relatively small size of our population, that was probably insufficient to detect a statistically significant difference. As a matter of fact, about 60% of TNBCs considered for this study measured ≥2 cm and our results are similar to previous data in literature (63% [21]).

For what concerns functional techniques, the mean ADC values were significantly higher for TNBCs than for non-TNBCs; even if it was not possible to establish a cut-off value, given the small study population, our data confirm the few pieces of evidence reported in previous studies [14,15]. Such difference in ADC values was suggested to be due to the presence of necrosis, that determines a fall in tumor cellularity with consequent increased diffusion [28].

Our study demonstrated that certain MRI features are significantly associated with TNBC on a 3 T scanner, such as circumscribed margins, rim enhancement and the presence of intralesional necrosis. On the contrary, irregular mass shape and not circumscribed margins, resulted more frequently associated with non-TNBC. Regarding functional imaging, the mean ADC values were significantly higher for TNBCs than for non-TNBCs. Nevertheless, we have several limitations. First of all, it is a monocentric, retrospective analysis, including a relatively small number of patients; therefore, results need to be further validated in larger population prospective studies. However, the number of patients included in other similar studies was not much bigger or even lower in some cases [7,12,13,16,17,20]. Second, the molecular subtypes of breast cancer were determined using immunohistochemical surrogates that lack in standardization, compared to gene profiling, even if they have shown similar clinical significance and are nowadays routinely used [40]. Finally, this study did not include a long-term evaluation of possible prognostic implications of MRI features associated with TNBC.

In the last decade researchers have demonstrated that different human cancer phenotypes show specific imaging texture features [41] and several authors have suggested that radiogenomic markers are able to distinguish among molecular subtypes of breast cancer [42,43,44,45,46]. Our objective for the future is to implement techniques of radiogenomic analyis in our research, to validate these promising results.

## 5. Conclusions

Our study suggests that TNBC is associated with specific features at MP-MRI that can be predictors of immunohistochemical and pathological results, with a consequent prognostic value. Further studies are needed to evaluate the possible impact of these findings on patient management in clinical practice.

## Figures and Tables

**Table 1:** MRI features. Statistically significant *p*-values are bolded.

	TNBC	Non-TNBC	*p*-Value
Number of Patients	26	24	
Tumor size			0.308
≥2 cm	15 (57.7%)	11 (45.8%)	
<2 cm	11 (42.3%)	13 (54.2%)	
Enhancement			0.192
Mass	24 (92.3%)	22 (91.7%)	
Non-mass	2 (7.7%)	2 (8.3%)	
Shape (masses)			**0.005**
Round	9 (37.5%)	3 (13.6%)	
Oval	5 (20.8%)	0	
Irregular	10 (41.7%)	19 (86.4%)	
Margins (masses)			**0.001**
Circumscribed	14 (58.4%)	2 (9.1%)	
Irregular/Spiculated	10 (41.6%)	20 (90.9%)	
Rim enhancement (masses)			**<0.001**
Yes	18 (75.0%)	2 (9.1%)	
No	6 (25.0%)	20 (90.9%)	
T2-signal intensity			0.301
Hyperintensity	6 (23.1%)	2 (8.3%)	
Isointensity/Hypointensity	20 (76.9%)	22 (91.7%)	
Intralesional necrosis			**0.016**
Yes	11 (42.3%)	2 (8.3%)	
No	15 (57.7%)	22 (91.7%)	
Perilesional edema			0.88
Yes	6 (23.1%)	5 (20.8%)	
No	20 (76.9%)	19 (79.2%)	
Multifocality/Multicentricity			0.064
Yes	13 (50.0%)	5 (20.8%)	
No	13 (50.0%)	19 (79.2%)	
Abnormal lymph nodes			0.262
Yes	16 (61.5%)	10 (41.7%)	
No	10 (38.5%)	14 (58.3%)	

**Table 2:** Logistic regression analyses. Statistically significant *p*-values are bolded.

	Univariate Analysis		Multivariate Analysis	
	OR (CI 95%) *	*p*-Value	OR (CI 95%) *	*p*-Value
Tumor size ≥ 2 cm	1.61 (0.53–4.93)	0.4	-	-
Enhancement			-	-
Mass	1.10 (0.14–8.42)	0.93		
Non-mass	1.44 (0.22–9.42)	0.7		
Shape (masses)			-	-
Round	3.0 (0.30–31.63)	0.4		
Oval	Out of scale	1		
Irregular	0.53 (0.06–4–32)	0.55		
Irregular/Spiculated margins (masses)	0.03 (0.01–0.17)	**<0.001**	0.03 (0.002–0.34)	**0.005**
Rim enhancement (masses)	29.86 (5.53–161.35)	**<0.001**	33.08 (1.59–687.55)	**0.02**
T2-signal intensity			-	-
Hyperintensity	0.22 (0.04–1.24)	0.09		
Isointensity/Hypointensity	1.20 (0.12–11.0)	0.89		
Intralesional necrosis	8.10 (1.56–41.72)	**0.01**	0.52 (0.02–11.04)	0.67
Perilesional edema	1.14 (0.30–4.37)	0.85	-	-

* OR = Odds Ratio; CI = Confidence Interval.
